# Head-Head Interactions of Resting Myosin Crossbridges in Intact Frog Skeletal Muscles, Revealed by Synchrotron X-Ray Fiber Diffraction

**DOI:** 10.1371/journal.pone.0052421

**Published:** 2012-12-20

**Authors:** Kanji Oshima, Yasunobu Sugimoto, Thomas C. Irving, Katsuzo Wakabayashi

**Affiliations:** 1 Institute for Protein Research, Osaka University, Suita, Osaka, Japan; 2 Division of Biophysical Engineering, Graduate School of Engineering Science, Osaka University, Toyonaka, Osaka, Japan; 3 Department of Biological and Chemical Sciences, Illinois Institute of Technology, Chicago, Illinois, United States of America; National Institute for Medical Research, Medical Research Council, United Kingdom

## Abstract

The intensities of the myosin-based layer lines in the x-ray diffraction patterns from live resting frog skeletal muscles with full thick-thin filament overlap from which partial lattice sampling effects had been removed were analyzed to elucidate the configurations of myosin crossbridges around the thick filament backbone to nanometer resolution. The repeat of myosin binding protein C (C-protein) molecules on the thick filaments was determined to be 45.33 nm, slightly longer than that of myosin crossbridges. With the inclusion of structural information for C-proteins and a pre-powerstroke head shape, modeling in terms of a mixed population of regular and perturbed regions of myosin crown repeats along the filament revealed that the myosin filament had azimuthal perturbations of crossbridges in addition to axial perturbations in the perturbed region, producing pseudo-six-fold rotational symmetry in the structure projected down the filament axis. Myosin crossbridges had a different organization about the filament axis in each of the regular and perturbed regions. In the regular region that lacks C-proteins, there were inter-molecular interactions between the myosin heads in axially adjacent crown levels. In the perturbed region that contains C-proteins, in addition to inter-molecular interactions between the myosin heads in the closest adjacent crown levels, there were also intra-molecular interactions between the paired heads on the same crown level. Common features of the interactions in both regions were interactions between a portion of the 50-kDa-domain and part of the converter domain of the myosin heads, similar to those found in the phosphorylation-regulated invertebrate myosin. These interactions are primarily electrostatic and the converter domain is responsible for the head-head interactions. Thus multiple head-head interactions of myosin crossbridges also characterize the switched-off state and have an important role in the regulation or other functions of myosin in thin filament-regulated muscles as well as in the thick filament-regulated muscles.

## Introduction

Muscles relax when the interaction between actin and myosin is blocked by molecular switches on either or both the thin and the thick filaments in a sarcomere which is the smallest functional and structural unit of striated muscle. Although myosin filaments in smooth muscles and certain types of invertebrate striated muscles participate in the regulation of muscle contraction, the role of thick filament structure in the regulation of striated muscles of higher vertebrates, which are primarily controlled by Ca^2+^-binding to troponin-tropomyosin on the thin filaments, has not been clearly elucidated. A thorough knowledge of the structure of the thick myosin filaments in muscles is essential if its participation in inhibitory or regulatory mechanisms in contraction of higher vertebrate striated muscles is to be understood. The last few years have seen significant advances in structural studies of thick myosin filaments from various types of muscles under resting conditions by (cryo-)electron microscopy (cryoEM) and three-dimensional (3D)-image reconstruction using single particle approaches [1], [2]. Using an atomic structure of myosin molecule, these studies have revealed the structure of thick filaments to nanometer-scale resolution, suggesting that interactions between myosin heads resulting in the formation of a so-called “interacting head motif” are responsible for switching off the myosin molecules in vertebrate smooth muscles [3], invertebrate striated muscles with phosphorylation-dependent regulation such as tarantula [4], [5] and limulus [6] muscles and in scallop muscles [7] with Ca^2+^-dependent (dual) regulation. Recent studies showed that similar head-head interactions of myosin crossbridges occurred in isolated thick filaments from vertebrate fish skeletal [8] and cardiac striated [9], [10] muscles and also in heavy meromyosin (HMM) molecules (comprising of the two heads and part of the rod) from vertebrate cardiac and skeletal muscles when they were treated with blebbistatin, a known inhibitor of actin-binding and ATPase (catalysis of the hydrolysis of adenosine triphosphate (ATP)) activity of myosin molecules, although those muscles are not thought to be intrinsically regulated by the myosin molecules [11]. Although this interacting head structure is a plausible model for relaxation based on isolated myosin filaments, it has not been clearly proved whether or not this structure occurs in the native myosin filaments in higher vertebrate muscles.

For this purpose, a more objective approach to the study of myosin filament structure is to use x-ray fiber diffraction where the advantage is the ability to examine the native, intact myosin crossbridge array in muscle cells, but with the disadvantage that the interpretation of the data has to rely on modeling due to the lack of phase information, that is different from EM-image analysis [12]. X-ray fiber diffraction patterns from invertebrate insect flight muscle and vertebrate bony fish muscle in which the myosin filaments are perfectly arrayed in a simple hexagonal lattice provided highly crystalline data in a resting state. They have been analyzed by crystallographic approaches to find a structural model for the myosin filaments indicating that there might be a head-head interaction in resting intact myosin crossbridges on the filament backbone [13], [14]. On the other hand, in higher vertebrate striated muscles such as frog and rabbit skeletal muscle fibers that have been well studied, there is little firm evidence for such an interaction of myosin heads due to their inherent complexities. One difficulty with these muscles is that the myosin filaments are not simply ordered in the sarcomere as in bony fish muscle but arrayed in a hexagonal superlattice (a unit cell side having √3 times the side of the regular hexagonal lattice) with “statistical” disorders [12]. This causes a partial sampling of the superlattice type on the myosin-based layer lines in the x-ray diffraction pattern and modeling for the observed intensity data could not be performed in a straightforward way. Another complexity is that there are distinct perturbations from perfect symmetry of the myosin crossbridge array in such a way that the three-fold symmetric crowns in the 42.9-nm-basic period of the myosin filament are not related by exact helical symmetry [12]. Such perturbations of the myosin crossbridge array are a common feature of vertebrate striated muscles and do not appear in the myosin filaments of invertebrate muscles. Recent single particle EM-3D-image analysis of fish [8] and cardiac [9], [10] myosin filaments showed azimuthal perturbations of myosin crossbridges in addition to the axial perturbations; there are one step of ∼0° and two steps of ∼60° at three individual crowns (the crown-like ring of myosin heads in each 14.3-nm-axial repeat) within the 42.9-nm-period rather than three equal azimutal steps of 40° between next crowns as in the perfect helical filament. Furthermore, in higher vertebrate muscles, these perturbations occur in sub-regions along the whole filament so that the myosin filaments have a mixed structure of a regular, singlet repeating region of 14.3 nm and a perturbed, triplet repeating region of 42.9-nm-repeat (14.3 nm×3) along the filament [15], [16] (see below). This statistical superlattice structure and the mixed population of regular and perturbed filament structures have been the two main difficulties faced in precise modeling the myosin filaments against the observed x-ray diffraction data from higher vertebrate striated muscles [12]. Recently we developed a method to remove the effects of partial lattice sampling on the myosin-based layer lines in the x-ray diffraction pattern from live frog skeletal muscles to procure single filament transforms in the framework of the superlattice array [17]. We also established an x-ray analytical treatment of the structure consisting of the portions with singlet and triplet crown repeats along the filament [16], [17]. These developments make it possible to perform more trustworthy modeling of the structure of resting myosin filaments in intact frog skeletal muscle than previously obtainable under the conditions of full thick-thin filament overlap in the sarcomere.

Most recently, Luther et al. [18] first demonstrated by an EM tomographic approach that myosin-binding protein C (MyBP-C; C-proteins) molecules were organized regularly on the thick filament backbone with a similar axial repeat to that of myosin crossbridges in frog skeletal muscle. Although the detailed structure of the C-proteins in intact vertebrate striated muscles is still unclear, it seemed that part of the molecule is bound to and the other part projects outward from the filament backbone. However, whether or not the C-protein periodicity is identical to the myosin repeat has been a matter of debate [18]–[23].

In this article we confirm, using high-angular resolution x-ray meridional diffraction patterns from live frog skeletal muscles, that the C-protein molecules have a slightly longer periodicity than that of the myosin crossbridges. We have used these data to develop an improved model of the thick filament, now including C-proteins, using previously established techniques and using the atomic model of the myosin head with bound ATP-hydrolysis products, ADP (adenosine diphosphate) and Pi (inorganic phosphate) (a pre-powerstroke shape) in order to investigate the configurations of myosin crossbridges in live resting frog skeletal muscle with nearly full thick-thin filament overlap. Firm evidence is presented here that there exist interacting head structures in the myosin filaments, underlying the relaxed state of myosin filaments in thin filament-regulated muscles.

## Materials and Methods

### Intact Muscle Preparation

Bulfrogs (*Rana catesbeiana*) with a body size of ca. 10 cm (L)×5 cm (W) were purchased from an experimental amphibian animal supply company and maintained under temperature and humidity controlled conditions for no more than three days prior to use in experiments. Under these conditions, the animals do not require feeding. Sartorius muscles were dissected from the hind legs and used for x-ray experiments. They were mounted in plastic chambers with the pelvic bone connected to a force transducer and a tibial end to a clip. The chamber was continuously circulated with chilled frog Ringer’s solution (115 mM NaCl, 2.5 mM KCl, 1.8 mM CaCl_2_, 0.85 mM NaH_2_PO_4_, 2.15 mM Na_2_HPO_4_, pH adjusted to 7.2). After muscle was contracted isometrically by electrical stimulations to decrease the crystallinity in a controlled manner [16], [17], the sarcomere length of whole muscle was slightly stretched to ∼2.4 µm, just over the rest length to aid better orientation of myofilaments in muscle, adjusted using He-Ne laser diffraction. (We call here this length of whole muscles “full thick-thin filament overlap length”.).

Work on amphibians does not require approval from the institutional committee for animal experiments at our institution. Animals were euthanized by pithing followed by decapitation. These procedures are consistent with the fundamental guidelines for proper conduct of animal experiment of the Ministry of Education, Culture, Sports, Science and Technology in Japan and the recommendations of the American Veterinary Association for euthanasia in this species.

### X-ray Diffraction Measurements

X-ray diffraction patterns from live resting muscles were measured at a camera length of *ca*. 2.4 m by using collimated synchrotron x-rays with a wavelength of 0.150 nm on beamline 15A at the Photon Factory, Tsukuba, Japan [24]. The apparent size of the x-ray beam was 0.3 mm vertically and 0.5 mm horizontally at the specimen. The flux of x-ray beam was ∼10^11^ photons·s^−1^. Two-dimensional x-ray diffraction patterns were recorded with a storage phosphor screen (imaging plates: type BAS-III, Fuji Film, Tokyo, Japan) [25] in order to measure the first to the eleventh order myosin-based layer line reflections as described previously [16]. The x-ray diffraction data from four muscles were summed and averaged after applying a scaling by the total intensity of the diffraction pattern except for the strong equatorial region and the part around the beam stop [26]. The meridional x-ray diffraction measurements were performed with high-angular resolution using the third generation synchrotron x-rays on beamline 18ID at the Advanced Photon Source, Chicago, USA [27]. The beam size of x-rays with the wavelength of 0.1033 nm was 0.037 mm×0.19 mm and the flux of x-rays was ∼10^13^ photons·s^−1^. A high-resolution CCD detector [28] was used to record the diffraction diagrams at the camera length of 5.0 m. In this system the precise axial spacings of the clusters of peaks due to the C-proteins and myosin crossbridges on the thick filament could be determined by assigning the third myosin meridional reflection at rest 14.34 nm as an internal calibration (see below). All x-ray measurements on live frog muscles were done at the temperature of 10°C which was motivated by the report of Huxley et al. [29].

### Analysis of x-ray Diffraction Data

After the x-ray exposure, the imaging plates were scanned with an image reader (BAS 2000, Fuji Film, Tokyo) using a pixel size of 100 µm. Digital intensity data were analyzed on graphics workstations (O2, Silicon Graphics Inc., USA and Power MacIntosh G4, Apple Computer Inc., USA). After determining the origin of each image and correcting the inclination angle of each image, the four quadrants of the x-ray diffraction patterns were folded and averaged [16], [26]. The integrated intensities of the myosin-based layer lines in x-ray diffraction patterns from muscles at rest were measured by the same procedure as described previously [16]. Each of the intensities of narrow stripes on the layer lines indexed to the first to eleventh order of the basic period of 42.9 nm (referred to as M1, M2, –, M11) was plotted as a function of the reciprocal radial coordinate *R*. The sampling effects on the layer lines due to the hexagonal filament array of a superlattice type were corrected by applying the cut-off method to the cylindrically-averaged difference Patterson function [Δ*Q*(*r, z*)] [30] to remove the inter-filament crossbridge vectors as reported previously [16], [17]. The cylindrically averaged difference Patterson function [Δ*Q*(*r, z*)] can be calculated by Fourier transformation of the layer line intensities (except for the equatorial intensities) diffracted from cylindrically averaged fibrous structures such as muscles. Since the equatorial reflections are always crystalline reflections due to the hexagonal filament array in striated muscles, being attributed to the interference between the thin and the thick filaments in projection onto the equatorial plane, it is not useful for analysis of a single filament structure. The Δ*Q*(*r, z*) is the autocorrelation function of the deviation from the axial average of the electron density along the constituent filaments, and the peaks appearing on the Δ*Q*(*r, z*) map correspond to the vectors between the mass centers of the monomeric units in the filaments and/or the positions of the heavy molecules if they bind to the filaments. The Δ*Q*(*r, z*) function is used here as a method to obtain a single thick filament transform by cutting off the inter-filament interference vectors. The clusters of peaks of each meridional reflection in the x-ray diffraction patterns which were obtained at the Advanced Photon Source were analyzed using the FIT2D program (ESRF; http://www.esrf.eu/computing/scientific/FIT2D) and they were deconvoluted using Gaussian functions, providing their peak profiles and integrated intensities. The periodicity of C-proteins was determined accurately from the axial spacings of their higher order components (see below). The intensity components of the 42.9-nm-based reflections were used for modeling of the resting myosin crossbridge orientation. The modeling procedure was as described below.

### Energy Minimization of the Atomic Structure of a Myosin Head and Electrostatic Potential on the Surface of Myosin Crossbridges in ADP.Pi-bound State

**Figure 1:**
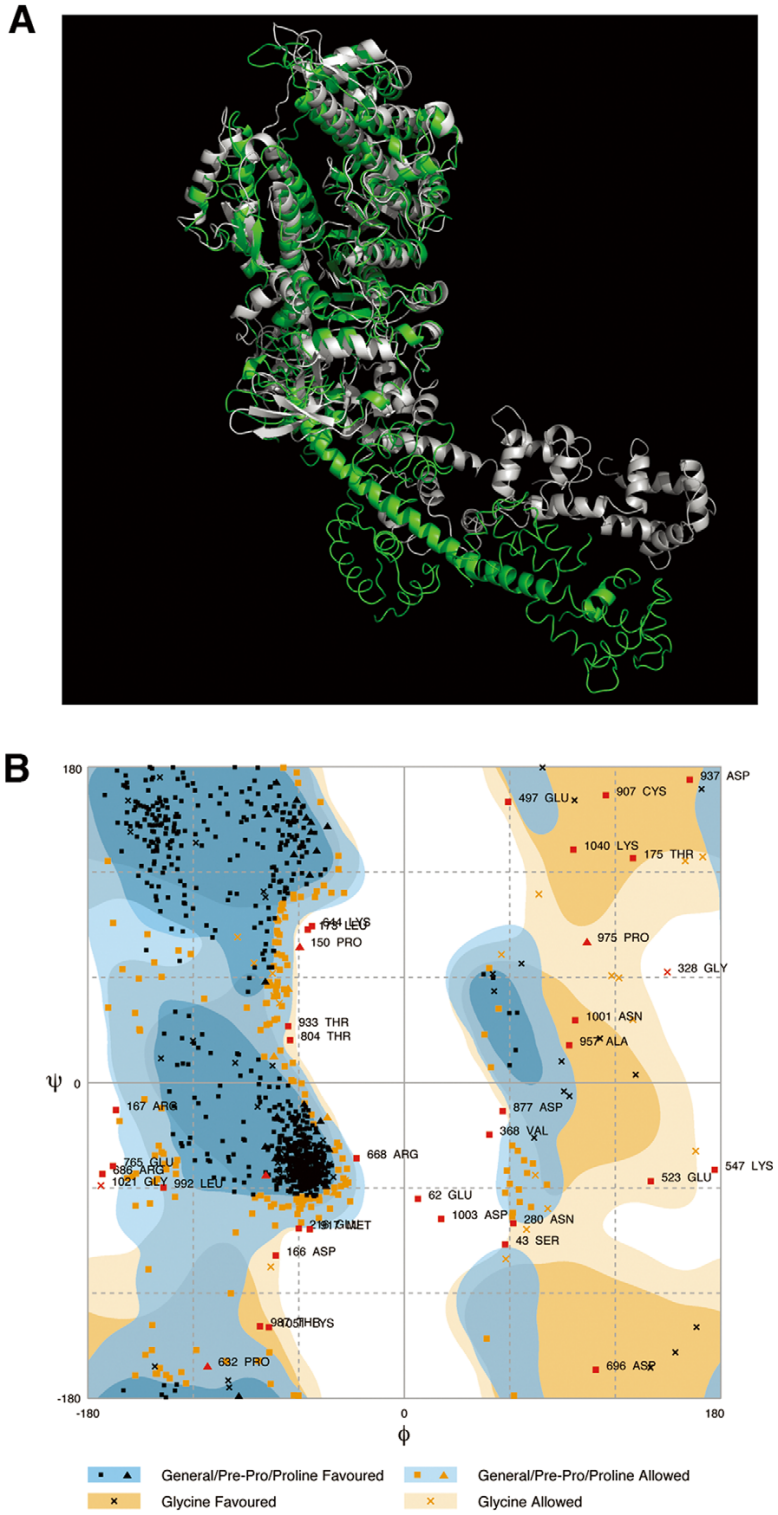
Atomic structure modeling of a myosin II S1. (A) Comparison of the atomic structures of myosin II subfragment 1 (S1) in nucleotide-free and ADP.Pi-bound states. Atomic structures of myosin II S1 in nucleotide-free state (green) and in ADP.Pi-bound (ADP.Pi) state (gray). The structure in nucleotide-free state was taken from PDBID: 2MYS [31]. The part of the motor domains of the S1 molecules is superimposed on each other. The secondary structure of the myosin heavy chain is diagrammed as a ribbon-and-wire cartoon. The radius of gyration of S1 (C°-model) is ∼4.56 nm in the nucleotide-free model and ∼4.45 nm in the ADP.Pi-bound model. (B) A Ramachandran diagram of S1 in the ADP.Pi-bound model. The diagram was generated using the CCP4 tool, rampage [36]. The ratios of the number of residues to the total number in favored regions, allowed regions and other (outlier) regions are 77.2%, 19.4% and 3.4%, respectively.

For atomic fitting studies, an atomic structure of skeletal myosin head (S1) (myosin II S1) in ADP and Pi-bound state (S1.ADP.Pi) (S1, a subfragment 1 part of a myosin molecule) was modeled using the 1072C°-model which was made by modifying the crystal structure of nucleotide-free S1 (PDBID: 2MYS) [31] to yield a better fit to the small-angle x-ray scattering profile of skeletal S1 in MgATP solution [32], [33]. Then the modified residues of N-dimethyl-lysine of the atomic structure of nucleotide-free S1 were replaced by lysine residues for estimating accurate electrostatic potentials on the surface of S1, but the missing chains in the crystal structure were not restored. The coordinates of main- and side-chain atoms except for C° atoms were modeled by “PULCHRA” (a tool for a full-atom reconstruction and a refinement of reduced protein models) [34]. Energy minimization of an S1.ADP.Pi structure in a vacuum was then done by using the “cosgene Molecular Dynamics (MD) engine of myPresto” [35]. The atomic structure of S1.ADP.Pi was superimposed onto the optimum 68-sphere model using the same parameters as shown in Table 1. Since the atomic structure was treated as a rigid body in superimposition, there were many steric clashes of side chains observed in the interface between the heads in the optimum orientation of a myosin crossbridge. Clashes were removed by further energy minimization. A Ramachandran plot was calculated to observe the validity of the atomic model. It is a way of validating an atomic model by seeing whether its C° atom’s dihedral angles of each amino acid are within realistic limits. There is the freedom of rotation about two bonds of amino acids allowing protein to fold in many ways. The rotations about these bonds can be specified by dihedral angles, called φ and ψ. Although there are many combinations of φ and ψ, a Ramachandran diagram visualizes their sterically favored, allowed and other (outlier) values of amino acids in protein as clustered regions on a two-dimensional plot. A structure and a Ramachandran diagram of the energy-minimized S1.ADP.Pi are shown in Fig. 1. This diagram was generated using the CCP4 tool, rampage by Richardson and coworkers [36]. For inspection of the nature of head-head interactions, electrostatic potentials on the surface of each head of the energy-minimized crossbridges were calculated by using the “APBS” tool in PyMOL (The PyMOL Molecular Graphics System, Version 1.5, Schrödinger, LLC.) [37].

**Table 1:** Summary of parameters of the optimum myosin filament models.

Parameters	Full-filament overlap model	Non-filament overlap model
*ε_r_* of a purple head (degree)	2	5
*ε_r_* of a red head (degree)	26	53
helical radius, *r_hr_* (nm)	9.8	11.2
*ε_p_* of a purple head (degree)	−13	9
*ε_p_* of a red head (degree)	15	354
helical radius, *r_hp_* (nm)	12.7	11.4
*φ_1_* (degree)	3	50
*φ_2_* (degree)	50	90
*φ_3_* (degree)	123	130

Parameters used to define the azimuthal orientation of myosin crossbridges on the thick filament in the model calculation. The first column identifies the parameters used in the modeling; *ε*, the rotation angle of a head about the *z*-axis and *r_h_*, the helical radius of a head where subscripts (*r*, *p*) denote those for the regular and perturbed regions, respectively. *φ*, “is the” azimuthal rotation angle of a myosin crossbridge in the perturbed region where subscripts (1, 2, 3) correspond to crown levels 1, 2 and 3 in Figs. 6B and H. The second and third columns identify the parameter values for the full-filament overlap and non-filament overlap models, respectively. The paired heads of a single myosin crossbridge are denoted as a red head and a purple head.

## Results

### X-ray Diffraction Patterns from Live Resting Frog Skeletal Muscles with Full Thick-thin Filament Overlap

**Figure 2:**
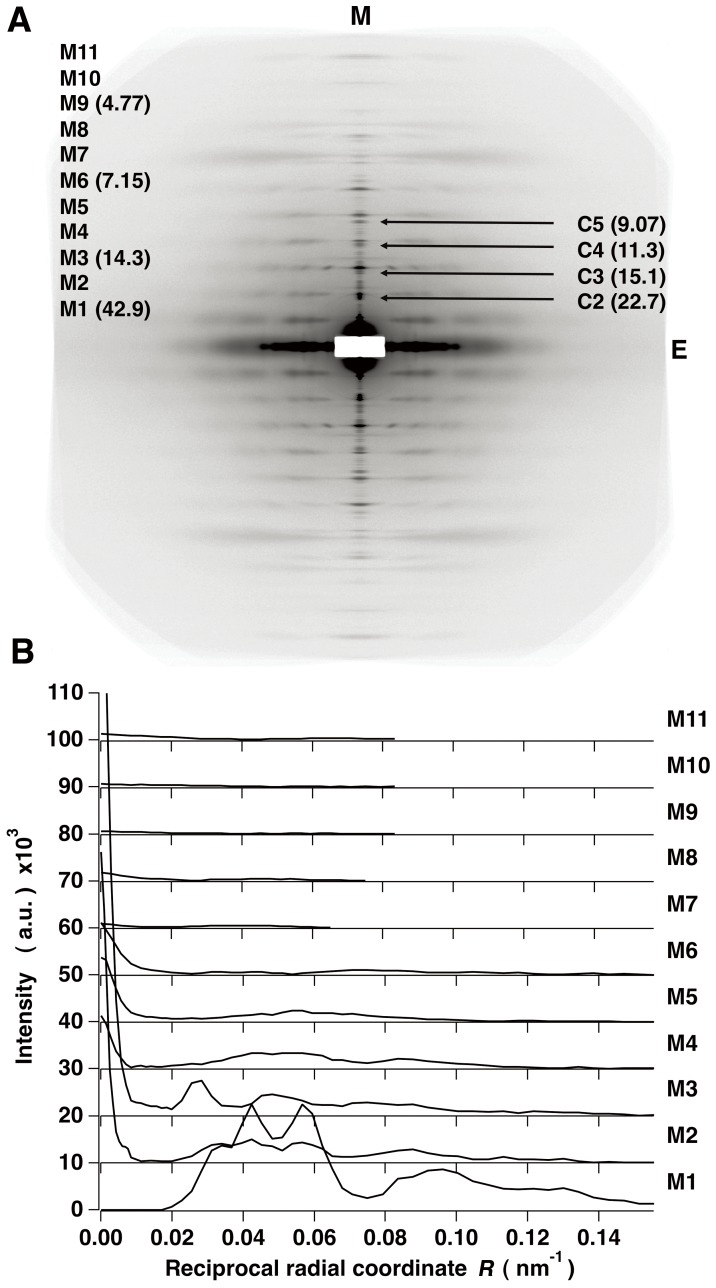
X-ray diffraction patterns from resting frog skeletal muscles. (A) An x-ray diffraction pattern in the resting state of live frog skeletal muscles with full thick-thin filament overlap, obtained at the Photon Factory. The background intensity is subtracted. The fiber axis is vertical. M1 to M11 denote the first to eleventh order myosin-based layer lines with a basic period of 42.9 nm. C2 to C5 denote the clusters of meridional reflections that may be assigned as the second to fifth order C-protein-associated meridional reflections with a period of 45.3 nm (see text). The values in parentheses are the axial spacing in real space (in nm). M, the meridional axis and E, the equatorial axis. (B) The observed intensity distributions on the M1 to M11 layer lines.

Figure 2A shows a low-angle x-ray diffraction pattern from live frog skeletal muscles at full thick-thin filament overlap length of a sarcomere in the resting state obtained at the Photon Factory (see Materials and Methods), in which the background intensity was removed. It shows a strong series of myosin-based layer lines at successive orders of a crystallographic period of 42.9 nm (denoted by the letter M). The dominant feature of the myosin pattern is a ladder-like appearance that is attributed to the three-stranded quasi-9/1 helices of thick filaments with the different myosin crossbridge crown separations within the 42.9-nm-period [16]. The x-ray diffraction pattern in Fig. 2A was obtained from muscles in which the strong hexagonal sampling by the superlattice filament array had been reduced in advance by applying repeated electrical stimulations [17]. Such muscles still gave residual sampling peaks of a superlattice type on the low-angle myosin-based layer lines, retaining a superlattice character and also showed the radially narrowed myosin meridional reflections (see Fig. 2B). The observed lateral widths of the myosin meridional reflections in these muscles corresponded to an apparent coherent diffracting length of ∼100 nm (∼one superlattice unit cell size). This size of a unit cell was utilized for modeling studies (see below).

### The Periodicity of MyBP-C (C-proteins) Bound on the Thick Myosin Filament Backbone

**Figure 3:**
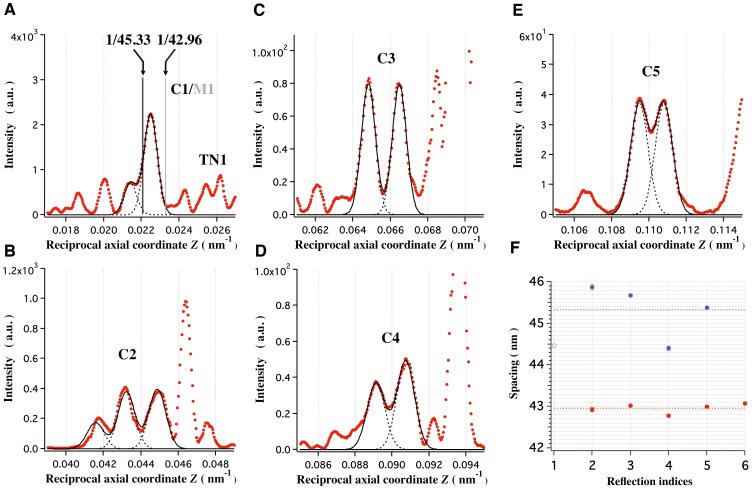
Axial intensity profiles of the C-protein-associated meridional reflections. The x-ray diffraction pattern was obtained at the Advanced Photon Source. (A) Those of the C1/M1 region. (B–E) Those of the clusters of the C2 to C5 meridional reflections. The experimental intensity profiles are denoted by red dotted curves and the fitted Gaussian functions are shown by black curves. (F) Axial spacings of each C-protein and each myosin reflection when divided by their reflection index. They are shown by blue and red full circles, respectively. Bars on the circles are the standard deviation of the mean from four data sets. The average period of C-protein is 45.33±0.58 nm and that of myosin is 42.96±0.11 nm. In (F), the spacing (44.5 nm) of the reflection which was enhanced by labeling antibody to C-protein [40] is shown by the blue open circle. In (A), there are triplet of reflections in 0.0205 nm^−1^< *Z*<0.0245 nm^−1^ with a main peak at 1/44.5 nm^−1^, and the axial positions of the reflections assigned as C1 and M1 are depicted by vertical lines. TN1 denotes the sampled first order troponin-based reflection.

Analysis of the myosin crossbridge arrangement around the thick filament backbone in resting muscle is the main concern of this paper and consequently, it is important to establish whether or not myosin binding proteins (MyBP) make a significant contribution to the myosin-based intensities. The myosin-based meridional x-ray diffraction pattern includes diffraction features due to structures other than myosin crossbridges such as C-proteins, titin/connectin and others in the thick filaments [15], [16], [38], [39]. According to Luther et al. [18], C-proteins align along the thick filament with three-fold symmetry at every third myosin crown level, and the part of the molecule is bound to and the other part is projecting outward from the thick filament backbone in frog skeletal muscle. However, it has not been resolved unambiguously whether or not C-protein has the same periodicity as myosin. The most intense of the meridional reflections in the x-ray diffraction pattern is a reflection at the spacing of ∼44.5 nm in the C1/M1 region (see Fig. 3A). Since the intensity of this reflection was considerably enhanced by labeling with antibodies to C-protein, it is at least partly caused by C-protein [40]. From inspection of a clustering of only this region, it has currently been suggested that C-protein has a somewhat longer repeat than myosin [39], [41]. However, in our previous work [16], it was claimed that the first order myosin reflection (M1) and first order C-protein reflection (C1), which are both sampled by interference effects between opposite halves of the A-band (the thick myosin filament-containing part) in the sarcomere, overlapped and that this overlaying might distort the positions and intensities of both reflections. Additionally, the C1/M1 region is affected by the form factor (Fourier transform) of the thick filament backbone with a finite length [15], [16]. Therefore, it was suggested that a more precise estimate of the C-protein periodicity should be made from its higher order meridional reflections, where cleaner diffraction features are present. In the unprecedentedly high-angular resolution meridional x-ray diffraction patterns obtained at the Advanced Photon Source (see Materials and Methods), we observed weak but clear clusters of reflections in groups which do not lie in positions ascribable to the myosin and actin filaments [16]. These clusters of reflections sit just at the low-angle side of each of the strong myosin-based reflections and might have been assigned as due to C-proteins (and partly other proteins) from their diffraction features at rest, during isometric contraction [16], [38], [39] and in rigor (the state after ATP-depletion). Figures 3A–E show the axial intensity profiles of these clusters of reflections (referred as C2–C5) including those of the C1/M1 region. After deconvolution of overlapping peaks by using Gaussian functions, each peak position of the clusters in the C2 to C5 regions was precisely determined and the axial spacings of the C2 to C5 reflections were estimated by intensity-weighted averaging of these clusters (Figs. 3B–E). These values fit to a single axial repeat, and the best average gave a periodicity of 45.33±0.58 nm. Similar analysis for the clusters of myosin-based reflections (M2–M6) gave rise to a current value of 42.96±0.11 nm (Fig. 3F). The average period measured from the interference separations between the clusters of peaks in the C2–C5 regions was 660±70 nm, being close to the center-to-center distance (710–730 nm) between the two C-zones (the C-protein-bound regions on the thick filament, forming seven transverse stripes between the thick filaments within the A-band) in a single A-band of a sarcomere which was derived from the EM measurements [20], [21], [23], [41]. This fact provides further support to the notion that these reflection groups are the result of diffraction by C-proteins. Thus, we confirmed that the periodicity of C-protein is, in fact, longer than that of myosin and that the C-proteins occupy a large part of the perturbed region along the thick filament as the C-zone, affecting the myosin crown arrangement in that region. This result is consistent with the EM observations by Sjöström and Squire [19] and Squire et al. [21] that the striation patterns changed through the C-zone, showing there were two different axial repeats in the C-zone in the fine structure of the A-bands of cryo-sectioned higher vertebrate muscles.

The axial positions of the C1 and M1 reflections are depicted in Fig. 3A. In frog skeletal muscle, C-protein has a modular structure containing eleven subdomains, three of which have their C-terminus ends anchored on the thick filament backbone at 42.9-nm axial separations while the remaining eight (N-terminus regions including one specific motif) project out from the backbone and are directed toward an adjacent actin filament [18], [22]. It seems likely that this projecting outer portion, occupying ∼70% of the total mass of a C-protein molecule, generates a C-protein repeat longer than myosin if that portion could interact systematically and specifically with the neighboring actin filaments in the hexagonal lattice [21], [22]. Recent EM tomographic analysis (a 3D-reconstruction method from a tilt series of EM-images) by Luther et al. [18] revealed that a major density portion of C-protein at high radius, beyond myosin crowns (>25 nm) might be in contact with actin in neighboring thin filaments. (Note that projecting portions of C-proteins have not been visualized so far on isolated the thick filaments, presumably due to disordering in the absence of the filament lattice [9].) They showed that the C-protein peaks on the plot profile of the tomographic EM-images were uniformly spaced based on the myosin repeat. Although the difference we observed in the periodicity between myosins and C-proteins (∼2 nm) might be expected to be discernible by EM-tomography, it may be hard to observe the two periodicities in the averaged EM-images because the C-protein array would be subjected to considerable axial disordering by the specific interaction with the surrounding actin filaments having an unequal helical repeat in the lattice [18]. More detailed studies on C-proteins in frog skeletal muscle will be reported elsewhere (manuscript in preparation). Briefly, our modeling studies showed that a series of the 45.3-nm-based meridional reflections were reproduced much better by including the C-protein structure with a periodicity of 45.3 nm in the thick filament model. Specifically, with C-protein left out from the model, the meridional reflections which may be assigned as the C-protein-associated reflections disappeared. As mentioned above, both myosins and C-proteins contribute to the meridional reflection at ∼1/44.5 nm^−1^ in the C1/M1 region and the observed intensity profile in this region was reproduced in the thick filament model including C-proteins with a periodicity of 45.3 nm more accurately than in the model having C-proteins with the same periodicity as myosin.

As for titin/connectin, it has an array of pseudo-repeating eleven domains with the same axial repeat as the myosin crossbridge array and may have an effect in locating C-protein at the level of every third myosin crown level through the N-terminal domains [9], [18], [39], but it is unclear whether titin/connectin molecules conform to the symmetry of the crossbridge array.

### Sampling-corrected Myosin-based Layer Line Intensities in x-ray Diffraction Patterns from Muscles with Full Thick-thin Filament Overlap

**Figure 4:**
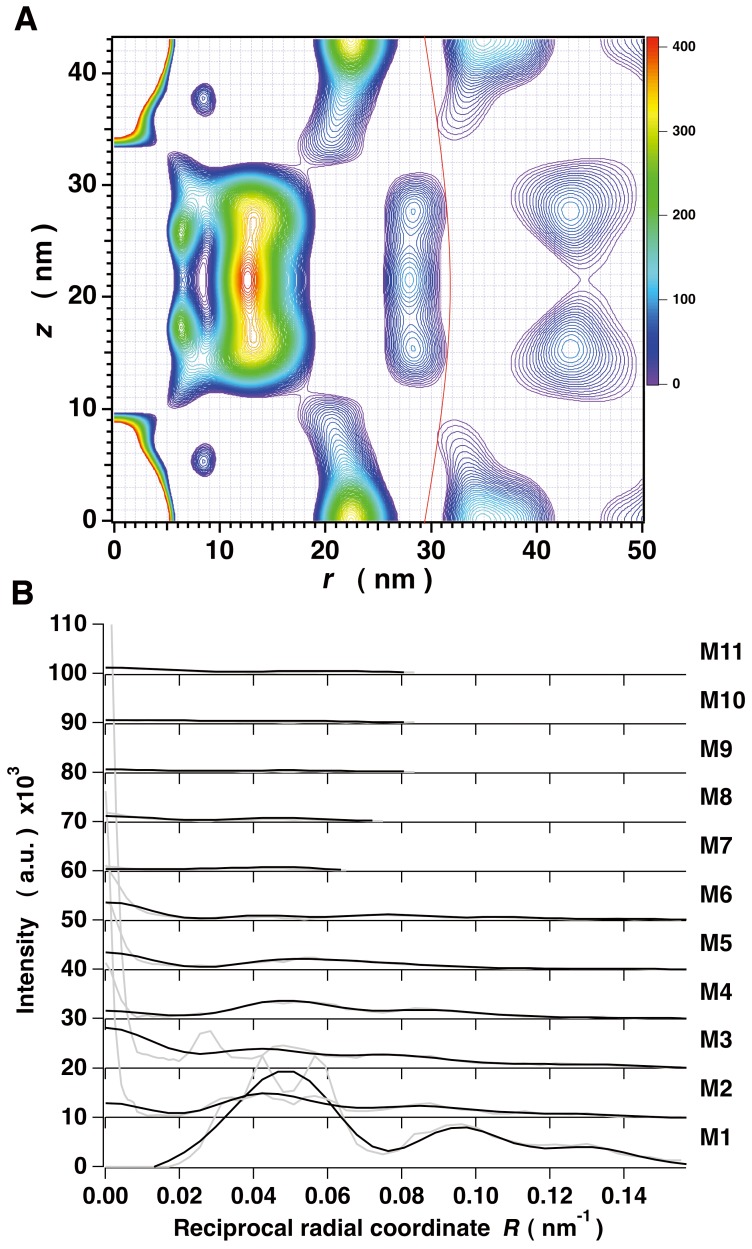
The cylindrically-averaged difference Patterson function and the corrected layer line intensities. (A) The *r*-*z* map of the cylindrically-averaged difference Patterson function [Δ*Q*(*r, z*)] calculated from the observed M1 to M11 layer line intensities. The map is contoured at levels between 0 and 410 at the intervals of 4.1. Only positive peaks are shown and negative peaks are omitted. The borderline (a red curve) is drawn distinguishing the region of inter-crossbridge vectors within a single filament from that of inter-filament crossbridge vectors. (B) Comparisons of the intensities (black curves) corrected by the cut-off method and the original layer line intensities from full-overlap muscles (gray curves). The layer line intensities are normalized so that the sum of the intensities except for the region close to the meridian (*R*<0.0254 nm^−1^) is identical between the corrected and original intensities (see text).

In order to eliminate residual lattice sampling effects on the myosin-based layer lines in the x-ray diffraction pattern, we have applied the cut-off correction (see Materials and Methods) to the cylindrically-averaged difference Patterson function [Δ*Q*(*r, z*)] calculated from the observed M1 to M11 layer line intensities as previously described [16], [17] (Fig. 4A). The Δ*Q*(*r, z*) map showed distinct inter-filament crossbridge vectors residing outside the borderline (indicated by a red curve) that distinguishes the region of primarily inter-crossbridge vectors within a single filament from the region of inter-filament crossbridge vectors as reported previously [17]. The positive peaks outside the red curve in the map reveal that thick filaments are still packed in a hexagonal superlattice form just as in unstimulated muscles. Inside the red curve, the lobe around (*r*, *z*) = (0 nm, ∼8 nm) indicates that the two heads of a myosin crossbridge are axially separated by ca. 8 nm. From the peak at (*r*, *z*) = (∼22.4 nm, 0 nm) the average of the helical radii (*r_h_*) of myosin crossbridge’s centers of mass is estimated to be ∼12.9 nm from the relation *r* = 2*r_h_* sin[π((*z*/C) +1/3)] (C; a pitch of the long-period helical strand (129 nm (42.9 nm×3)). This value was used as an initial radius of the crossbridges in the optimum search for the azimuthal orientation of myosin heads around the filament backbone (see below). As was done previously [17], we cut off the peaks in the region outside the red curve in the Δ*Q*(*r, z*) map to procure the layer line intensities of single filaments by Fourier-Bessel transformation of this truncated Δ*Q*(*r, z*) (the “cut-off correction”).

Figure 4B compares the corrected layer line intensities with the original data. *RDI* (see below) calculated between them was ∼0.199. The corrected layer lines showed much less sampling peaks indicating that the cut-off correction reduced the lattice effects on off-meridional parts of the layer lines to an insignificant level. However, the contribution of residual interference due to what we have called “face-to-face” inter-filament crossbridge vectors (corresponding to those between the filaments facing in opposite directions) still affected the intensities in the region close to the meridian (*R*<0.0254 nm^−1^); a detailed examination on this point was made in the previous paper [17]. Therefore the intensity data of *R*<0.0254 nm^−1^ were not used for modeling the azimuthal orientation of myosin crossbridges.

### Optimum Modeling for Azimuthal Orientations of Two-headed Myosin Crossbridges Around the Filament Backbone

**Figure 5:**
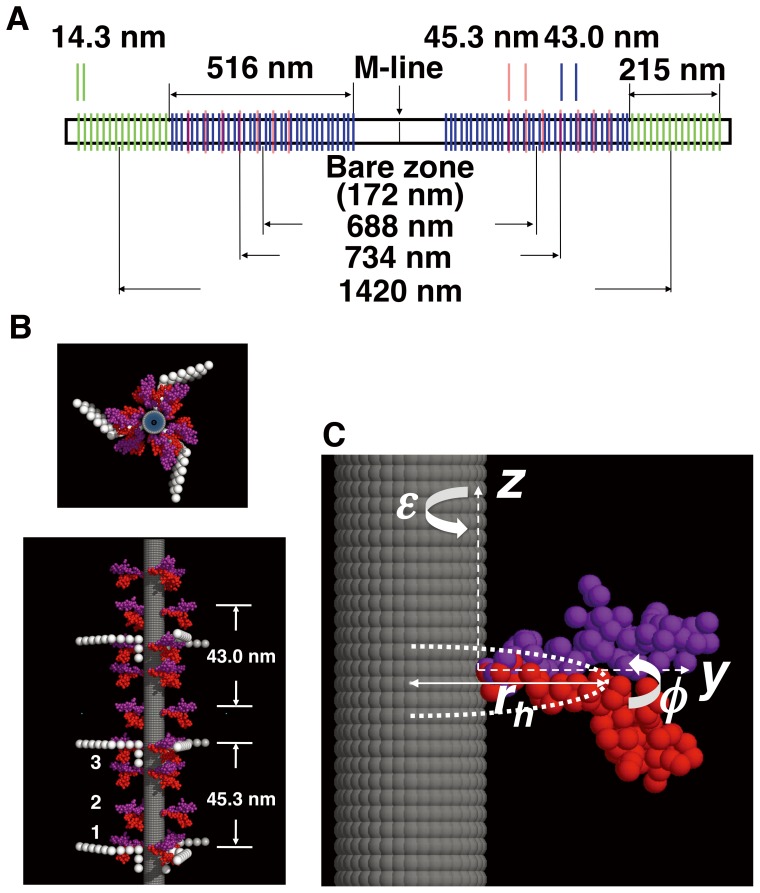
Modeling crossbridge structure of the frog skeletal thick myosin filament. (A) Distributions of the perturbed region (blue), the regular region (green) of myosin crossbridge arrays and the C-protein region (red) along the thick filament in resting frog muscles. (B) Overview of the thick filament model including C-proteins. Members of paired heads constituting a single myosin crossbridge are distinguished by a red or purple color. C-protein is bound to the thick filament backbone every at the level of crown 1. Eleven domains of a C-protein having a molecular weight of ∼130 kDa are shown by white spheres, each having a diameter of 4 nm [18]. The backbone of the myosin filament is shown as a gray cylinder. Upper, a top view and lower, a side view. (C) Parameters describing the arrangement of a two-headed myosin crossbridge in terms of a 68-sphere model on the filament backbone in ADP and Pi-bound state. The *z*-axis is parallel to the filament axis. *φ* is the rotation angle of crossbridges about the filament axis and *ε* is the rotation angle of the two heads of a crossbridge about the *z-*axis. *r_h_* is the average of helical radii of myosin crossbridges. Paired heads of a single crossbridge and the backbone are denoted as in (B).

An optimum search for the orientation of two-headed crossbridges was performed using the corrected off-meridional intensity data shown in Fig. 4B. As an initial model for resting myosin filament structure, we started with the mixed structure model with two different axial periodicities (i.e. the regular region and the perturbed region) along the filaments, which was derived by intensity analysis of the myosin-based layer lines including the meridional reflections in the x-ray diffraction patterns from resting live frog muscles reported by Oshima et al. [16]. In this model, the perturbed regions extended from the bare zone (the central region of thick filaments in the absence of crossbriges) end toward the Z line (the dense partition at each end of the sarcomere) in the sarcomere, occupying ∼70% of the whole length of a thick filament (∼516 nm) and the regular regions were located at the distal ends, occupying ∼30% of the filament length (∼215 nm) (Fig. 5A), similar to the model of Malinchik and Lednev [15]. EM studies have shown that C-proteins are located in the region which ranges from ca. 260 to 560 nm from the M-line (part crosslinking transversely the thick filaments at their midpoints) in half of the A-band and that the center-to-center distance between the two C-zones in the single A-band is 710–730 nm [20], [21], [23], [41], close to the present x-ray value. Assuming that the presence of C-proteins affects the myosin crossbridge arrangement along the thick filament [8]–[10], [23], we introduced the C-protein array overlaying on the perturbed region of the initial myosin filament model (see Fig. 5A). Since the accurate structure of C-protein itself is not known, the position and configuration of C-protein on the thick filament were based on the tomographic EM-model of Luther et al. [18]. The structure of C-protein, having a molecular weight comparable to that of S1, was modeled by eleven spheres with a repeat of ∼4 nm and a diameter of ∼4 nm. C-proteins with their projecting portion bent near its center were aligned assuming three-fold rotational symmetry and a repeat distance of 45.33 nm along the thick filament (Fig. 5B). Assuming that the accessibility of C-protein to sites on actin will be at different axial levels due to the incommensurate repeats of the thick and the thin filaments, axial disordering effects of the projecting portion of C-protein were taken into account by using the isotropic temperature factor [exp(−4π^2^ *σ* ^2^ *Z* ^2^)] (where 2*σ* is ∼2 or 4 nm) (see Discussion). Although the length of the perturbed region (∼516 nm) in the present model is longer than that of the C-protein region (∼300 nm) reported by Luther et al. [18], it may be possible that the presence of C-proteins and other non-myosin proteins continues to affect the myosin crown arrangement in the non-overlap proximal region ranging from ca. 80 to 260 nm from the M-line.

**Figure 6:**
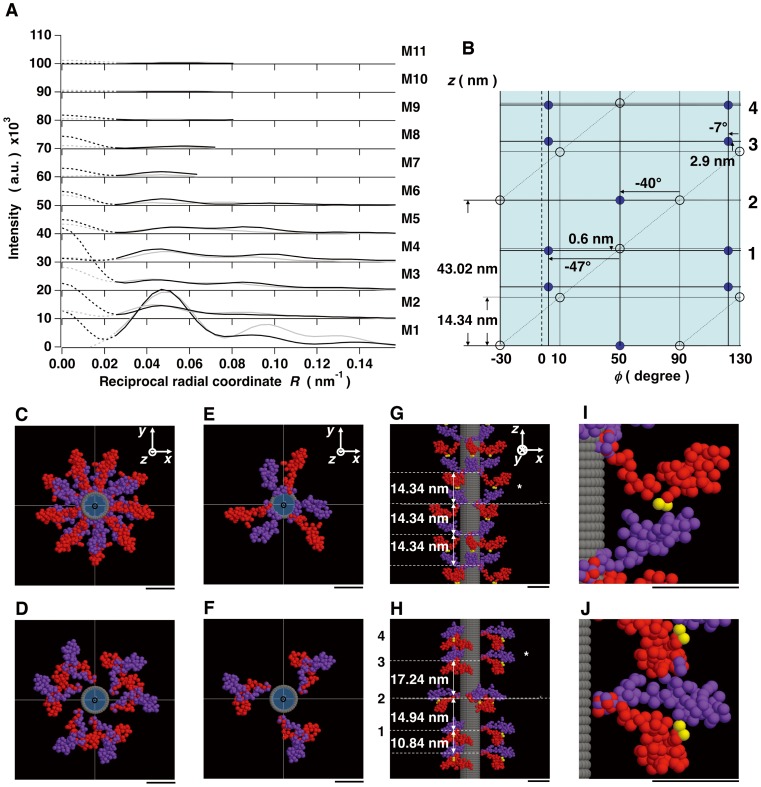
The corrected layer line intensities and the thick myosin filament model. (A) Comparison of the corrected layer line intensities (gray curves) from nearly full-overlap muscles and the intensities calculated from the optimum model with the azimuthal perturbations and C-proteins (the full-filament overlap model) (black curves). The intensities indicated by dashed lines in the region of *R*<0.0254 nm^−1^ are not used for modeling (see text). The intensities are normalized as in Fig. 4B. (B) Part of the radial projection of the crossbridge helices of a myosin filament in the perturbed region showing the azimuthal locations of crowns 1, 2, 3 and 4 and the displacements due to the axial and azimuthal perturbations. The abscissa axis is denoted by *φ* instead of 0∼2π*r*. Blue filled circles and black open circles denote respectively the shifted and regular locations of the crossbridge centers along the three 9/1 helical lines. Each dashed line shows each 9/1 helix in the three-stranded regular helical filament. (C) and (D) The full-filament overlap models in projection view in the regular (C) and perturbed (D) regions. (E) and (F) Those in cross-sectional view at the axial level of crown 2 in the regular (E) and perturbed (F) regions. (G) and (H) Those in side view in the regular (G) and perturbed (H) regions within the basic repeat. (I) and (J) Close-up views of the inter- and intra-molecular interactions between the myosin heads at the asterisk in (G) and (H). Yellow spheres mark the converter portion of the red head. The bottom of (G)-(J) is toward the M-line. The backbone is described as a structure-less cylinder. Scale bar, 10 nm.

For our modeling, the structures in the regular, perturbed and C-protein regions were treated individually as was done previously [16]; the transform of each of these three regions was calculated and added together to obtain the whole filament transform. The filament backbone was expressed as a featureless cylinder because it contributes little to the layer line pattern [42]. It is thought that the pre-dominant species of myosin heads in resting muscles are in the S1.ADP.Pi state [43]. An atomic structure of chicken skeletal muscle S1 has been reported in nucleotide-free state (PDBID: 2MYS) [31], which was used in our previous analysis [16]. For the present fitting studies, the atomic structure of S1.ADP.Pi (a pre-powerstroke structure) made from the 1072C°-model of S1.ADP.Pi [32], [33] (see Materials and Methods) was newly employed and superimposed onto the initial head model in which the atomic structure of nucleotide-free S1 was used [16]. In order to reduce computation time, each head of a single myosin crossbridge was assumed to have the same conformation (shape) in all regions (see below) and then approximated by 68 spheres with each of radius 0.72 nm. (A correlation coefficient of this sphere model against the 1072C°-model in Fourier transforms was 0.999 to the present resolution.) The axial orientation of each head against the filament axis was first optimized around the initial orientation to fit the meridional intensity profiles in high-angular resolution x-ray diffraction patterns as was done previously [16]. This optimization changed slightly but did not significantly alter the axial orientation of heads from the initial model. The positions and configurations of crossbridges in the regular and perturbed regions were allowed to be different since the myosin crossbridge structure in the perturbed region is thought to be affected by the interaction with C-proteins [8]–[10], [23] (see above). Fixing the structural arrangement of C-proteins as in Fig. 5B, we performed the optimum search for the azimuthal orientation of myosin crossbridges. Since the periodicity of C-protein is close to myosin, the transforms of the C-protein array on the thick filament backbone would partially overlap the myosin-based layer line intensities. It is profitable, therefore, to include the contribution of C-proteins in analysis (see Discussion). As mentioned above, in the perturbed region it was assumed that in the set of three successive crowns the crossbridge configurations are the same but their centers of mass deviate in the azimuthal direction without a change in the radial direction. In contrast, in the modeling of bony fish muscle [14], [44], the crossbridge configuration on the three crown levels was varied based on different density profiles along the filament axis from their EM-3D-image analysis [8]. Figure 5C illustrates the three variable parameters involved in the orientation search of myosin crossbridges about the filament axis; *ε* is the rotation angle of each of heads about the *z*-axis, which is parallel to the filament axis, passing through the innermost ends of a single crossbridge from which the two conjoined heads diverge, and *φ* is the azimuthal rotation angle of the two-headed crossbridge’s center of mass about the filament axis, and *r_h_* is its helical radius. *ε* was defined as zero at the disposition shown in Fig. 5C and allowed to vary from 0° to 360° in appropriate steps under the restriction that the myosin heads cannot direct toward the backbone. *φ* was set to be 90° as the angle of crown 2 of the 9/1 helix in the radial projection net (see below Fig. 6B) and its value was constrained so that any pair of *φ_1_, φ_2_* and *φ_3_* at three myosin crown levels within the 42.9-nm-period has the same angle or the value of *φ_3_−φ_1_* is at least 120° (see below). This took into consideration that both analyses by x-ray diffraction [45]–[47] and neutron diffraction with contrast variation [48] have revealed that the structure projected on the equatorial plane appears to have six-fold rotational symmetry, not nine-fold rotational symmetry at low resolution. *φ* was also allowed to locally vary around the initial value. *r_h_* was varied around 12.9 nm which was estimated from the peak position at (*r, z*) = (22.4 nm, 0 nm) in the Δ*Q*(*r, z*) map (see above). Finally, all of the parameters were optimized to obtain the best fit of the calculated intensities to the observed data. The calculated layer line intensities were derived with the same grid as that of experimental results. The optimum was determined by searching for a model giving the lowest value of following *RDI* (the residual deviation of intensity) as defined previously [17]:
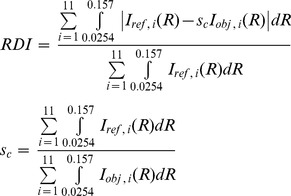
(1)where *I_ref, i_*(*R*) is the *i*th order layer line intensity of the reference, *I_obj, i_*(*R*) is that of the objective and *s_c_* is a scale factor between them. The integration excludes the data in the region close to the meridian (*R*<0.0254 nm^−1^) for the reasons mentioned above. Disordering effects of myosin crowns were not taken into consideration here. With the inclusion of atomic structure of S1.ADP.Pi, this modeling analysis may achieve nanometer-scale resolution.

Figure 6A compares the corrected M1 to M11 layer line intensities with the calculated ones from the final optimum model. The intensities derived from the improved model including the C-protein arrangement and azimuthal perturbations of the myosin crossbridge arrangement, yielded a better agreement (*RDI*∼0.30) with the observed intensities than those from the model without them (*RDI*∼0.39) (data not shown). This result confirms that in resting frog muscle, the azimuthal perturbations of myosin crossbridges occur in addition to axial perturbations as seen in bony fish [14] and cardiac muscles [9], [10], and that there was a small influence of C-protein for the myosin-based layer line intensities (see Discussion). Hereafter, this optimum model of a thick filament in nearly full-overlap muscle is referred to as “the full-filament overlap model”. Figure 6B shows the part of the radial projection [12] of the quasi-helical arrangement of myosin crossbridges in the perturbed region of the thick filament (looking outward from the central axis toward the surface of the backbone) in the full-filament overlap model. Note that the rotation angle (*φ*) of a crossbridge about the filament axis shown in Fig. 5C is taken as the horizontal axis in Fig. 6B instead of 0∼2π*r* (*r*, radial coordinate). The axial separations between the three crowns within the 42.9-nm-period were ∼10.84 nm, ∼14.94 nm and ∼17.24 nm as reported previously [16]. There was a large gap between crowns 2 and 3. The azimuthal angles of three crowns (crown 1, crown 2 and crown 3) were ∼3°, ∼50° and ∼123° (Table 1) where the myosin crossbridges at crown 3 and at adjacent crown 4 (or crown 1) had the same angle but the crossbridges at crown 2 had a different angle. The azimuthal aspects of perturbations in crossbridge orientations in different crowns in frog muscle may be slightly different from those found in bony fish muscle [8], [14]. Each of the angular separations between individual crowns was ∼47° or ∼73°, not far from 60°, ensuring that the structure of the thick filament in projection down the filament axis has pseudo-six-fold symmetry (see below). Figures 6C and D show projection views down the filament axis of one basic period in the regular and perturbed regions, respectively. The structure of the thick filament had nine-fold rotational symmetry in the regular region, reflecting the perfect three-stranded 9/1 helices with three-fold rotational symmetry. On the other hand, in the perturbed region the projected structure of the thick filament had apparently pseudo-six-fold rotational symmetry. The helical radius of the crossbridge’s center of mass in the full-filament overlap model was ∼9.8 nm in the regular region and it was ∼12.7 nm in the perturbed region, i.e. greater than that in the regular region. This appearance may be attributed to the fact that the regular region lacks C-proteins and is located at the distal ends of the thick filament in the resting state, where the backbone of the thick filament is tapered toward the Z-band [2], [41]. Since the diameter of the filament backbone in the uniform portion is ∼14 nm [12], the relatively smaller helical radius of crossbridges in both regions indicates that the S2 portions (subfragment 2 of HMM) lie close to the filament backbone. Figures 6E and F show cross-sectional views of the azimuthal orientations of myosin crossbridges around the filament axis at the axial level of crown 2 in the regular and perturbed regions, respectively. The distal ends of paired heads of a myosin crossbridge were oriented in different directions, more so in the regular region than in the perturbed region. Each head of a single crossbridge in each of the regular and perturbed regions had a different orientation about the filament axis. In the regular region, the helical radii of purple- and red-colored heads in a pair were ∼8.7 nm and ∼12.0 nm, respectively. In the perturbed region, they were ∼13.7 nm and ∼11.7 nm, respectively. In the regular region, the two heads of a single crossbridge flared, forming a “windmill-like” head-pair shape. In the perturbed region, one head of a crossbridge lay close axially on the partner head in a pair at the same crown level and came in contact with each other, forming a “cross-shaped” head-pair structure. The nearest distance between the surfaces of these two heads was less than 0.1 nm, indicating that the intra-molecular interactions between the paired heads of a single crossbridge occur at this interface (see Fig. 6J). Figures 6G and H show side-views of the configurations of myosin crossbridges in the regular and perturbed regions, respectively. In the regular region, one head seemed to be in contact with the neighbor head on the upper crown level along the filament axis (see Fig. 6I). The nearest distance between the surfaces of these two heads was about 0.5 nm, and it is possible that relatively weak inter-molecular interactions between the heads of axially adjacent crossbridges occur at this interface. In the perturbed region, one head seemed to make contact with the neighbor head in the axially adjacent crown only in the closest inter-crown separation (Fig. 6J). The nearest distance between the neighbor heads was less than 0.1 nm, also indicating the occurrence of inter-molecular interactions between these heads at this interface. It is clearly shown that the x-ray model had the resting heads projecting out from the filament surface rather than lying down flat. The structural parameters of the full-filament overlap model are summarized in Table 1.

### Nature of Head-head Interactions in Resting Frog Skeletal Myosin Filaments

**Figure 7:**
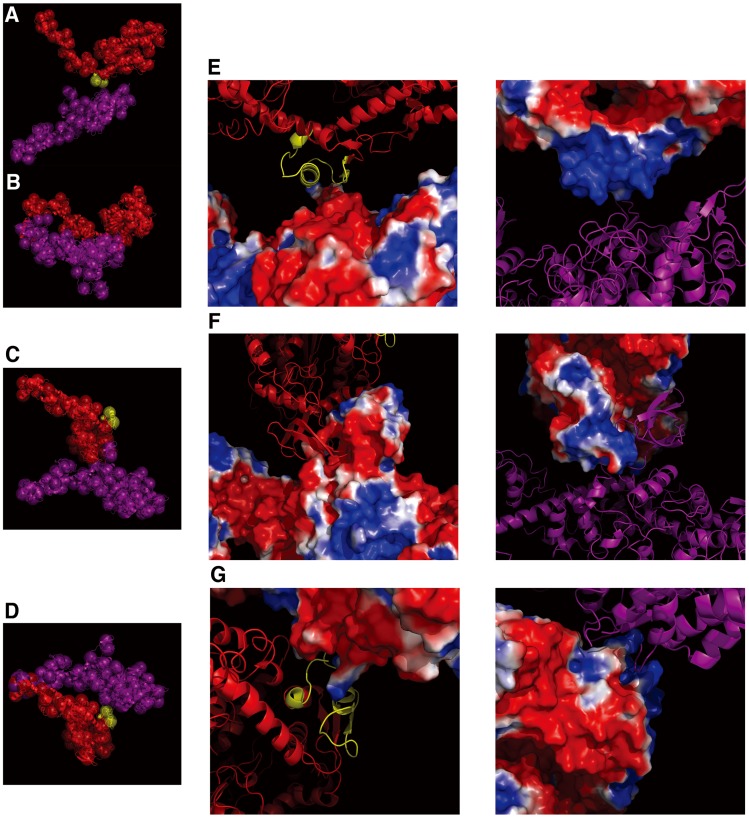
Inter- and intra-molecular interactions between heads of myosin crossbridges. The directions of the view are same as in Figs. 6I and J. The converter domain (residues 724–764) is marked by a yellow color. The secondary structure of the myosin heavy chain is shown as in Fig. 1A. (A) The inter-molecular head-head interactions in the regular region. (B) An atomic structure model of a tarantula myosin crossbridge (PDBID: 3DTP) [5] without the S2 structure and by its 75-sphere model made in the same way as in the case of the frog sphere model. A head (red) in tarantula myosin is put with the same orientation of the overall structure onto a myosin head (red) in the regular region of the full-filament overlap model. In both (A) and (B), the blocking head is toward the converter domain of the adjacent head. (C) The inter-molecular interactions and (D) the intra-molecular interactions between heads in the perturbed region. (E) - (G) Close-up views of electrostatic potential on the molecular surface of the myosin heads in the inter-molecular interactions of the regular region in (E) and those in the inter- and intra-molecular interactions of the perturbed region in (F) and (G), respectively. The negatively and positively charged regions are shown by red and blue colors, respectively, in the range of −1∼1 k_b_T/e (k_b_, Boltzmann constant; T (310K), absolute temperature; e, electron charge). For the charge distribution, one of myosin heads is diagrammed as a ribbon-and-wire cartoon and the other head is shown as a molecular surface.

Recently, fitting of atomic structure models to cryoEM-3D-image reconstructions of isolated tarantula (invertebrate) thick filaments under resting conditions by single particle analysis suggested that there were both intra-molecular and inter-molecular interactions between the two heads of individual myosin molecules in phosphorylation-regulated myosin filaments (PDBID: 3DTP) [5]. Our full-filament overlap model of the frog skeletal myosin filament was compared to the tarantula model. Note that the tarantula myosin filaments do not appear to have perturbed regions. Although the myosin crossbridges in the regular region of the frog model made inter-molecular head-head interactions between the crossbridges at adjacent crown levels whereas those in the tarantula model made intra-molecular head-head interactions (what was named as “J”-like motifs by Craig and his colleagues [2], [4]) in a single crossbridge as also seen in the EM of smooth muscle HMM crystal [3]. The interface region between the two interacting heads in the two models, however, was very similar to each other (Figs. 7A and B). A portion of the lower 50-kDa-domain on the head (purple) seemed to block part of the converter domain (yellow) of the neighbor head (red) as seen in tarantula myosin [5]. On the other hand, myosin crossbridges in the perturbed region appeared to make not only inter-molecular head-head interactions but also intra-molecular head-head interactions (Figs. 7C and D). A portion of the 25-kDa-domain of one head (red) appeared to direct toward the convex side of the neighbor head (purple), which consists mostly of the 25-kDa-domain, making inter-molecular head-head interactions (Fig. 7C). Simultaneously, a portion of the upper 50-kDa-domain of one head (purple) of a single crossbridge appeared to block part of the converter domain of the partner head (red), making intra-molecular head-head interactions (Fig. 7D). The interface region between the two heads was different from that between the heads in the regular region. In frog muscle, a J-motif configuration was not an obvious feature in the head-head interaction motif.

In order to investigate the nature of the head-head interactions in frog skeletal myosin filaments, the electrostatic potential distributions on the molecular surface of myosin heads were calculated (see Materials and Methods). In the regular region, an interaction area of the “blocking” head was negatively charged while the area of the converter domain of the neighbor head was positively charged (Fig. 7E). Similarly, in the perturbed region, one of interaction areas of the heads both in the inter- and intra-molecular interactions was positively charged and the other was negatively charged (Figs. 7F and G). In addition, the inter-molecular head-head interaction areas in the perturbed region seemed to be complementary in shape. These appearances suggest that electrostatic interactions play a predominant role for both of the inter- and intra-molecular head-head interactions in resting frog myosin filaments.

## Discussion

This article aims to provide structural information concerning resting crossbridge arrangement around the myosin filament backbone in typical higher vertebrate striated muscles that may lead to a detailed understanding of their participation in regulatory and/or modulatory mechanism(s) influencing force generation during muscle contraction. The myosin filaments in these muscles have a mixed population of regular and perturbed regions of myosin crown repeats along the filament axis [15], [16]. They are arranged in a statistical superlattice in the sarcomere, producing a quasi-crystalline sampling on the myosin-based layer lines in the x-ray diffraction pattern [12], [49]. We have already established an analytical approach for the mixed structure of myosin filaments in frog skeletal muscle [16]. Although the spatial distribution of the thick filament orientations in the sarcomere lattice has implications for a distinct role in muscle function [49], [50], the presence of the mixed structure has made this type of structural analysis very complicated, constituting a barrier to the rigorous elucidation of the myosin filament structure. To avoid the partial lattice sampling problem, frog muscles stretched beyond the overlap between the thin and the thick filaments in the sarcomere have often been used under the assumption that the myosin filament structure remains the same as in full-overlap muscles because they give rise to x-ray diffraction patterns with less sampling on the layer lines [15], [16], [51]. However, it is known that stretching does not completely remove the lattice sampling effects on the layer lines, and whether or not the filament structures in non-overlap and full-overlap muscles are really the same is not clear (see below). Here, using the atomic model of myosin II-S1.ADP.Pi (a pre-powerstroke head structure), only the intensity data from full-overlap muscles were analyzed and detailed configurations of resting myosin crossbridges without the potential confounding effects of stretch were elucidated.

### The Use of the x-ray Diffraction Data from Muscles with Full Thick-thin Filament Overlap

**Figure 8:**
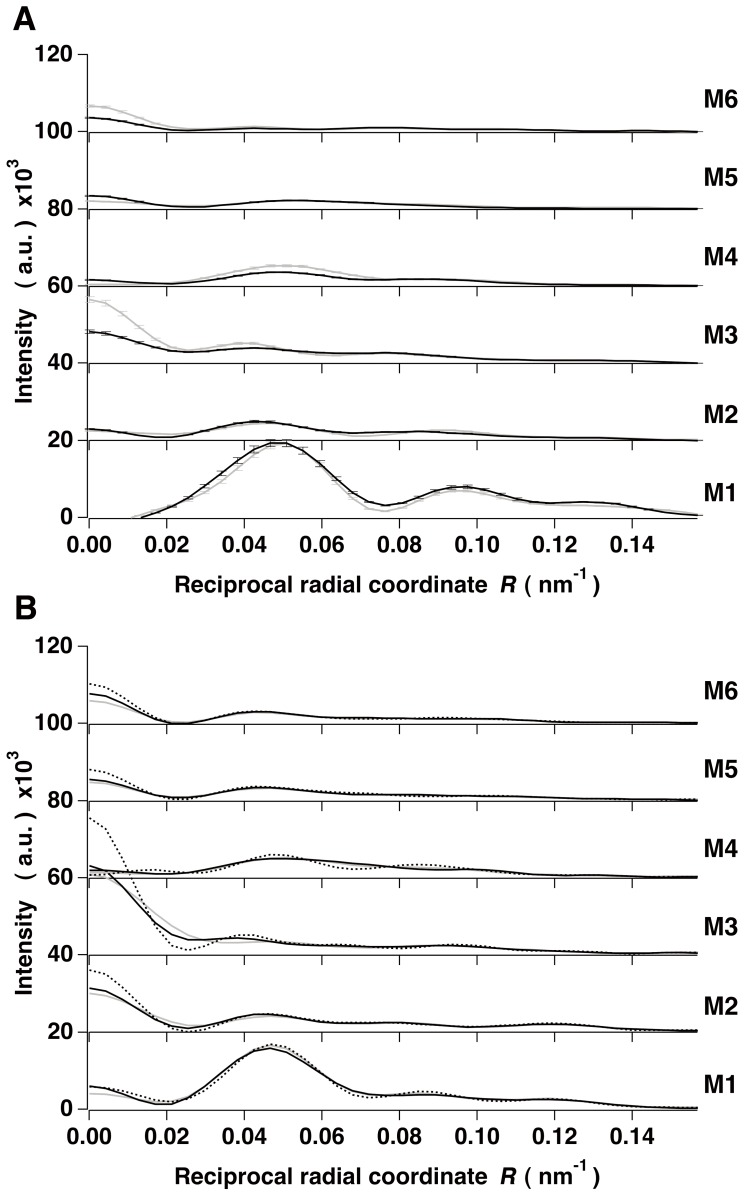
Layer line intensities from full- and non-overlap muscles obtained after the cut-off correction and their models. (A) Comparison of the corrected M1 to M6 layer line intensities from full-overlap (black curves) and non-overlap muscles (gray curves). The intensities are normalized as in Fig. 4B. (B) Comparison of the corrected layer line intensities of the hexagonal filament-array model with a filament spacing of ∼34.4 nm (black dotted curves), those with a filament spacing of ∼43.2 nm (black curves) and those of the single filament model (gray curves). Bars on the layer line intensity curves are the standard deviations of the mean from four data sets. The average value of the standard deviations of mean is ∼1.2% which was used as an acceptable window (see Fig. 11).

When the intensities of the myosin-based layer lines in non-overlap (overstretched) muscles (semitendinosus) with a filament spacing of ∼34.4 nm after the cut-off correction had been applied were compared with the present data in full-overlap muscles (sartorius) with a filament spacing of ∼43.2 nm, we found small but significant differences between them in the intensities of several layer lines (Fig. 8A). (Note that sartorius muscle is difficult to stretch to the sarcomere length where no overlap between the thin and the thick filaments occurs, and semitendinosus muscles are used for overstretch.) The *RDI* (denoted as *RDI_o_*) calculated between them was ∼0.171 in the range of *R*>0.0254 nm^−1^. We have investigated the source of this discrepancy using modeling studies. We set up models of the thick filament without C-protein for the non-overlap muscle and the full-overlap muscle in which the myosin filaments having the same structure as the non-filament overlap model (see Table 1) were arranged in one unit cell of “no three-alike” (packing rules of thick filaments in the lattice) superlattice [49] with the filament spacing of ∼34.4 nm and ∼43.2 nm, respectively. (The reason why C-protein was not included in these models is that its effects on the myosin-based layer line intensities are small (see below)). The *RDI* between the corrected layer line intensities derived from both models (denoted by *RDI_m_*) was ∼0.127 (Fig. 8B), being significantly smaller than the *RDI_o_* (Fig. 8A). As demonstrated previously by Oshima et al. [17], this was largely because the so-called “face-to-face” inter-filament crossbridge vector components which were not excluded by the cut-off correction contaminate the region inside the borderline in the Δ*Q*(*r, z*) map (Fig. 4A). These components generate continuous transforms on the layer line intensities, their contribution increasing as the filament spacing is shorter [17]. Thus, the effect becomes much greater in non-overlap muscle than in full-overlap muscle. The greater value of *RDI_o_* than *RDI_m_* indicates that the myosin filament structure of non-overlap muscles is probably not the same as that of full-overlap muscles. In the modeling studies which were done previously using the intensity data from non-overlap muscles (semitendinosus) [16], the myosin crossbridges in the regular region become oriented to make similar inter-molecular head-head interactions to those seen in the full-filament overlap model, but the crossbridges on the same crown level in the perturbed region become oriented to form a “flared” head-pair structure, different from a “cross-shaped” head-pair structure in the present full-filament overlap model. (Molecular parameters obtained for non-overlap muscles are given as reference in Table 1.) This difference constitutes a large part of the difference between the *RDI_o_* and *RDI_m_*. Therefore, it should be better to use the corrected layer line intensities from full-overlap muscles alone than using those from non-overlap muscles for modeling the azimuthal orientation of myosin crossbridges about the filament axis. Apart from this, it seems certain that the head-head interactions are a common feature of higher vertebrate muscles, independent of the degree of overlap between the thick and thin filaments in the sarcomere.

### The Contribution of C-proteins to the Myosin-based Layer Line Intensities

As mentioned above, the periodicity of C-proteins bound to the thick filament backbone as a whole was larger than that of myosin crossbridges. A larger periodicity of C-protein could be produced by interaction with actin in the adjacent thin filaments in the A-band lattice of a sarcomere [22], [39], and this interaction may be responsible for the set of seven stripes within the nine in the C-zone in each half of the A-band seen in the EM photographs of longitudinal-sectioned muscles [18]–[21], presumably contributing to the resting ordering of the myofilaments in the A-band lattice of vertebrate striated muscles.

**Figure 9:**
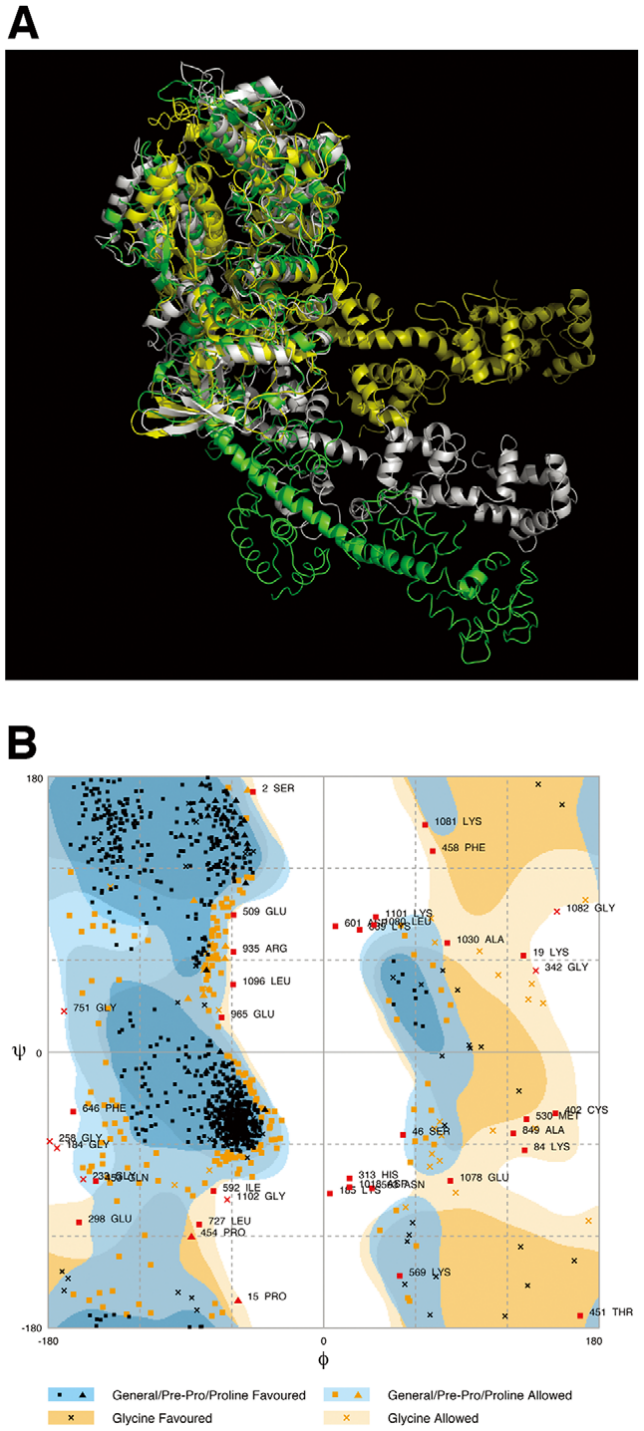
An atomic structure model of a myosin II S1 made by a homology modeling. (A) The atomic structures of myosin II subfragment 1 (S1) in ADP.Pi-bound state (yellow) made by a homology modeling method (cf. Fig. 1A) (see text). S1 molecules in nucleotide-free and ADP.Pi-bound states are shown by green and gray colors, respectively. The radius of gyration of S1 (C°-model) is ∼4.66 nm in the homology model. Each part of the motor domains of three S1 molecules was superimposed on each other, where the models shown by green and grey colors are the same as in Fig. 1A. The secondary structure of the myosin heavy chain is shown as in Fig. 1A. (B) A Ramachandran diagram of S1 in the homology model. The diagram was generated using the CCP4 tool, rampage [36]. The ratios of the number of residues to the total number in favored regions, allowed regions and other (outlier) regions are 78.1%, 18.5% and 3.4%, respectively.

**Figure 10:**
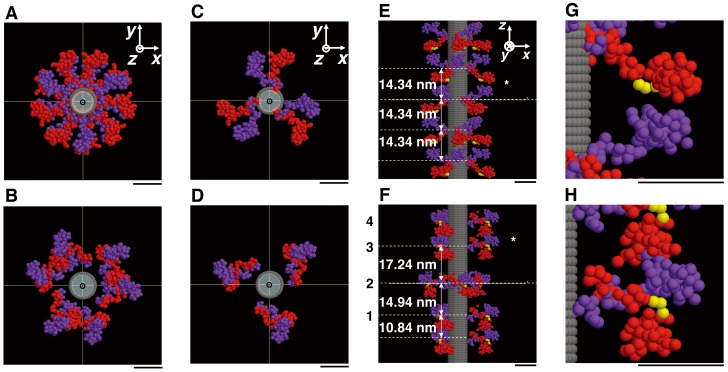
A frog skeletal myosin filament model using the homology model of S1. (A) and (B) Optimum models of the homology model of S1 in projection view down the filament axis in the regular and perturbed regions, respectively. The optimization was done in the same way as described in the text. (C) and (D) Those in cross-sectional view at the axial level of crown 2 in the regular and perturbed regions, respectively. (E) and (F) Those in side view within the basic repeat in the regular and perturbed regions, respectively. (G) and (H) Close-up views of the inter- and intra-molecular interactions between the myosin heads at the asterisk in (E) and (F), respectively. Yellow spheres mark the converter portion of one head. The bottom of (E) – (H) is toward the M-line. The filament backbone is described as a structure-less cylinder. Scale bar, 10 nm. The orientations of myosin heads in the homology model are very similar to those of the S1.ADP.Pi model in the text (Figs. 6C–J). The *RDI* of this optimum model is ∼0.32, slightly higher than that of the full-filament overlap model.

Since both periodicities are close to each other, the transforms of the C-protein arrangement would overlap partially with the myosin-based layer lines. We investigated the contribution of C-protein to the myosin-based layer line intensities using the full-filament overlap model including the C-protein array overlaying on the crossbridge perturbed region as shown in Fig. 5B. Then the cylindrically averaged layer line intensity [*I*(*R*, *Z*)] can be written as a sum of the intensities of a myosin component, a C-protein component and their cross-term,
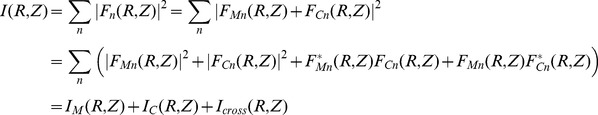
(2)in which *F_Mn_*(*R*, *Z*) and *F_Cn_*(*R*, *Z*) are the transforms of myosin crossbridges and C-proteins on the thick filament, respectively and F_Mn_*(*R*, *Z*) and F_Cn_*(*R*, *Z*) are their conjugate complexes, where the suffix *n* denotes the order of the Bessel function of the first kind contributed to the layer lines, and *I_M_*(*R*, *Z*), *I_C_*(*R*, *Z*) and *I_cross_*(*R*, Z) are the intensities of the respective components and the cross-term (i.e. the interference term between myosin crossbridges and C-proteins), respectively. The two intensity components and their cross-term were calculated and their contribution to the myosin-based layer lines was estimated as the ratio against the sum of their components. Each of the intensities of C-protein and cross-term components had a small effect on the region close to the meridian (∑*I_C_*/∑(*I_M_*+*I_C_*+|*I_cross_*|) ∼ 0.044 and ∑|*I_cross_*|/∑(*I_M_*+*I_C_*+|*I_cross_*|) ∼ 0.070 for *R*<0.0254 nm^−1^) and little effect on the layer line region (∑*I_C_*/∑(*I_M_*+*I_C_*+|*I_cross_*|) ∼ 0.008 and ∑|*I_cross_*|/∑(*I_M_*+*I_C_*+|*I_cross_*|) ∼ 0.029 for *R*>0.0254 nm^−1^) when 2*σ* in the temperature factor for axial disordering effects was 2 nm (see above). Incidentally, if the periodicity of C-protein is the same as myosin, the ratio of the sum of *I_C_* and |*I_cross_*| against the total sum is ∼ 0.230 for *R*<0.0254 nm^−1^ and ∼ 0.072 for *R*>0.0254 nm^−1^; the intensities of the myosin meridional reflections are markedly affected but the off-meridional layer line intensities are less affected by C-proteins. The C-protein effects come to be negligibly small on the myosin reflections when 2*σ* in the temperature factor was ∼ 4 nm [18].

**Figure 11:**
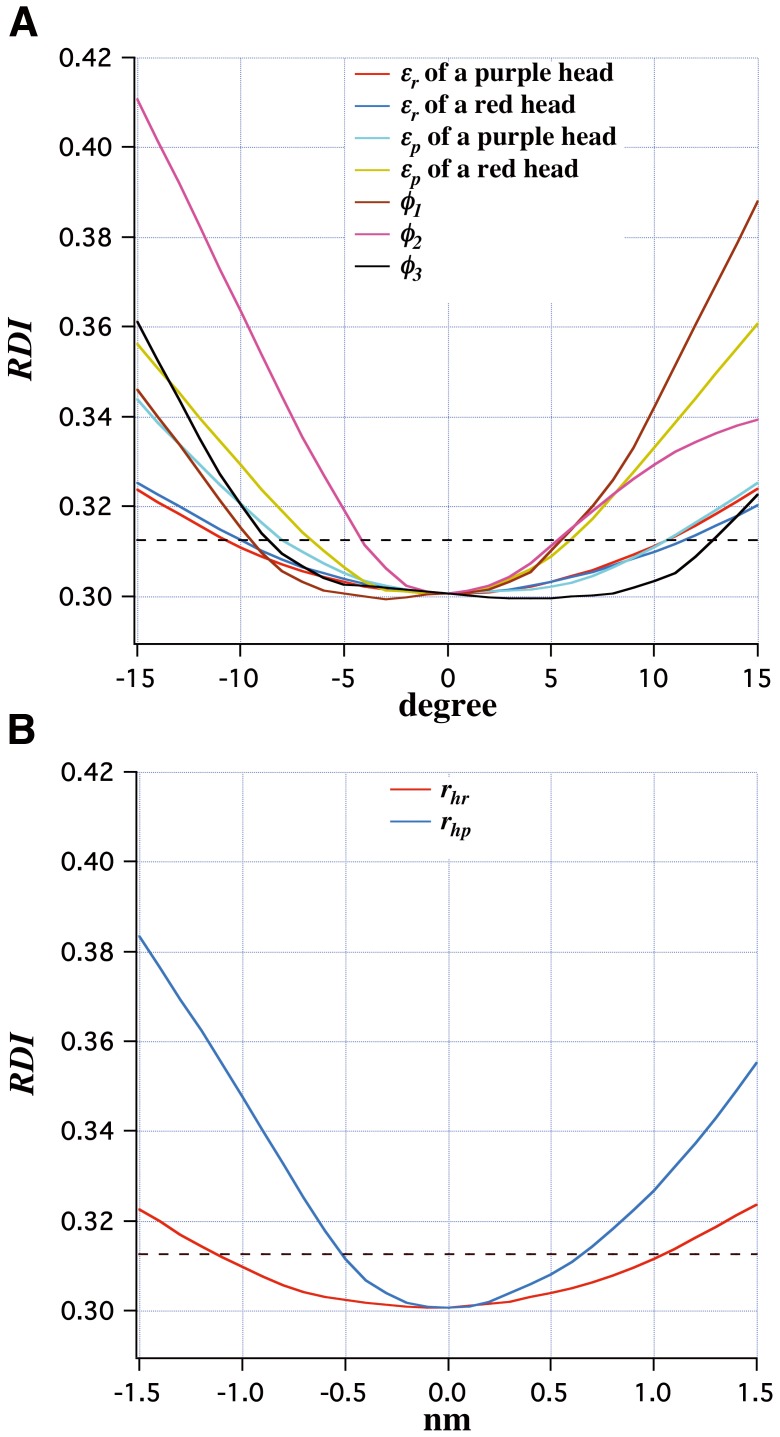
The sensitivity of the various head parameters in the full-filament overlap model to small shifts. (A) The changes in *RDI* (equation 1) against the rotations of a crossbridge around the *z*-axis in the regular (*ε_r_*) and perturbed (*ε_p_*) regions and the azimuthal rotations (*φ*) of crowns 1, 2 and 3 in the perturbed region. (B) The changes in *RDI* against the shifts from the optimum helical radii of the crossbridges in the regular (*r_hr_*) and perturbed (*r_hp_*) regions. Horizontal broken lines are drawn at the level of a ∼1.2% acceptable window above the minimum value of *RDI* of the full-filament overlap model.

### Azimuthal Perturbations of the Myosin Heads along the Thick Filament in Resting Frog Skeletal Muscles

In the perturbed region of the optimum filament model, the azimuthal (rotation) angles of heads of a myosin crossbridge at three crowns (crown 1, crown 2 and crown 3) within the 42.9-nm-period were ∼3°, ∼50° and ∼123° (Fig. 6B), giving rise to pseudo-six-fold rotational symmetry in the structure of the thick filament in projection down the filament axis (Fig. 6D). The myosin crossbridges at crowns 3 and 4(1) had the same azimuthal angle and the crossbridges at crown 2 had a different angle. Such an overall appearance was similar to the azimuthal perturbations of myosin crossbridges shown by x-ray analysis of bony fish muscles [14], so they do not depend on whether or not muscle has a simple lattice or a superlattice. Since the perturbed region occupies ∼70% of the whole filament length, the structure of the thick filament projected onto the equatorial plane would be dominated by pseudo-six-fold rotational symmetry. Thus, the presence of azimuthal perturbations may be closely related to a six-fold symmetric appearance of the thick filaments in frog skeletal muscle as revealed by equatorial x-ray diffraction [45]–[47] and neutron diffraction analyses [48] where the six spokes of myosin head density projected into the spaces between pairs of adjacent actin filaments. The fact that this kind of perturbations is seen in the EM-images of isolated myosin filaments [8] indicates that the non-helical head distribution is an intrinsic property of the striated muscle thick filaments *per se* but may not be produced due to lattice effects.

### Electrostatic Interactions between the Heads of Myosin Crossbridges in Resting Frog Skeletal Muscles

As mentioned above, it seems likely that the helical arrangements of myosin crossbridges around thick filament backbones in resting frog muscles are stabilized by two kinds of interactions between the heads of individual crossbridges, leading to the well-ordered x-ray diffraction pattern from the thick filaments in the resting state. The inter-molecular head-head interactions occur in the regular region where electrostatic interactions operate between the motor domain portion of the blocking head and the converter domain portion of the neighbor head. In the perturbed region, there are, in addition, intra-molecular interactions between the heads due to electrostatic interactions operating here between a portion of the upper 50-kDa-domain of the blocking head and part of converter domain of the partner head in a pair, envisaging the potential for cooperative interactions in regulation. In the inter-molecular interaction, electrostatic interactions operate between the 25-kDa-domain portion of one head and the same domain in the neighbor head with a complementary shape. Thus, the interactions between the heads are primarily electrostatic, and the converter domain is responsible for the head-head interactions characterizing the switched-off state. Kinetic studies have reported that the converter domain modulates the kinetic properties of myosin ATPase in solution and in muscle fibers [52]. Our x-ray analysis also reveals that the converter domain may have a key role for the regulation or inhibition mechanism of actomyosin interactions in resting vertebrate muscles. Further studies will be needed to clarify whether or not there are some distinct differences in the head-head interactions between in muscle and in isolated thick filaments.

### Myosin Crossbridge Structures in Various Resting Muscles

Head-head interactions have been revealed in various types of striated muscles [2], [12]. In the case of insect flight muscle, inter-molecular head-head interactions appear to be between adjacent myosin molecules on the same crown level [13]. In other invertebrate striated muscles (tarantula, scallop and limulus) they appear to be between the heads on the same and different crown levels [4]–[7]. Interactions of heads on the same crown level form a “J-motif” as mentioned above [4]. The head conformations or shapes are similar in these invertebrate muscles in which the myosin filaments lack a perturbed region of crown repeats and are arranged in a simple hexagonal lattice. Interactions between heads on different crown levels yield continuity of myosin head density along the long-pitched helical strands of these myosin filaments. In vertebrate bony fish muscles in which the myosin filaments are arrayed in a simple hexagonal lattice, Al-Khayat et al. [8], [14] did not differentiate between regular and perturbed regions along the myosin filaments in their modeling, thus generating a mean image covering both regions. The crossbridge organization and each conformation of two heads of crossbridges on each of the three crown levels within the basic repeat are different (conformation perturbation), and the paired heads of a single myosin crossbridge interact with each other on the two levels, lying one above the other and the heads on the third level lie side by side [8]. In their final model the head-head interactions on two of three crown levels within the basic period revealed the Wendt-type configuration of the interacting motif as seen in the EM-image of HMM crystal [3], [14]. As mentioned above, our best-fit model of the thick filaments in frog muscles revealed that one head of a myosin crossbridge seemed to be in contact with the neighbor head on the axially adjacent crown level in the regular region and one head seemed to make contact with the other on adjacent crown only at the closest inter-crown levels in the perturbed region (Figs. 6G–J). In frog muscle, the interacting head motif was observed in the inter-molecular interactions whereas in bony fish muscle, it is seen in the intra-molecular interactions. It may be worth mentioning that in the tarantula model the intra-molecular head-head interactions appeared to form a “J-motif” between the heads, but in the frog model a “J-motif” configuration was not obviously observed in the head-head interactions. Recently Cooke and his colleagues have explored the notion that locking “J-motif” forms between myosin heads correlates to a “super-relaxed” state (a slow ATP-turnover state) for the resting myosin ATPase in mammalian muscles [53] and tarantula muscle [54]. However, the present work suggests that a strict “J-motif” formation between myosin heads is not necessarily the only option available for electrostatically governed head-head interactions that can lock down resting myosin head interactions and ATPase.

Although the head-head interactions and head organization in myosin crossbridges appear to depend on the specific muscle type, it seems certain that the interacting head-head structure is a common feature in myosin filaments which possibly is generated by a formation of the closed conformation of myosin heads inhibiting phosphate (Pi) release and an actomyosin interaction in resting striated muscles [55]. Such head-head interactions producing different head organization on the relatively uniform surface lattice may have a distinct role in defining the regulation or other functions of the myosin heads in various muscles [12].

In our modeling analysis, we confined ourselves to obtaining the minimum information regarding characteristic features of resting myosin crossbridges. Since we deal with continuous layer line intensity data of limited resolution, it is difficult to determine a unique model when the number of parameters to be fitted to the observed x-ray data is increased. For this reason and reduction of computation time, we did not vary the conformation (shape) between the two heads of any crossbridge or between the heads of crossbridges in the regular versus perturbed regions along the filament as mentioned above. In practice, our present modeling does not show a large difference between using the nucleotide-free structure of a head (S1) and using the ADP.Pi-bound structure of S1. Recently, a head model fitted to the resting tarantula myosin EM-3D-image reconstruction using an atomic model of smooth muscle S1 complexed with ADP.AlF_4_ [56] (AlF_4_; an analog of Pi) showed a much larger bend and twist in the hinge region of S1 [5]. A homology structure of skeletal S1 was modeled based on the free head of HMM in tarantula muscle (PDBID: 3DTP) [5] (Fig. 9). A pairwise sequence alignment of a skeletal S1 (PDBID: 2MYS) [31] and a tarantula S1 (a, c and e chains of a free head) in PDBID: 3DTP [5] (a, c and e chains are the motor domain including lever-arm, the essential light chain and the regulatory light chain, respectively) was first done with respect to each chain by a “MAFFTash” tool [57], [58] (a program for alignment of multiple sequences and structures). After the alignment, each structure of the chains in skeletal S1 was modeled based on the structure of the corresponding chain of tarantula S1 by the “MODELLER” tool [59] (a program for homology or comparative modeling of protein 3D structures). Finally, an overall structure of skeletal S1 was modeled by combining the structures of three chains and its energy minimization was done by “cosgene MD engine of myPresto” [35]. We then attempted to use this S1 structure in the modeling, revealing that the highly bent/twisted S1 shape did not influence significantly the main results of the present modeling, where the *RDI* was ∼0.32, slightly higher than using the ADP.Pi-bound structure of S1 (Fig. 10).

### Major Structural Parameters Contributing to the Myosin-based Layer Line Intensities

Our x-ray modeling reproduced the observed intensity data with a global minimum *RDI* of 0.30. This comparatively high value of *RDI* was mainly due to a poor fit of the first layer line between the calculated and observed intensities (see Fig. 6A). This poor fit did not largely depend on the models of S1 shape used in the present work. The observed intensity on the myosin-based first layer line beyond *R*∼0.08 nm^−1^ is unavoidably contaminated by the axially broad actin-based first layer line intensity [60]. In view of this limitation, the general features of the myosin crossbridge structures in our modeling were found to produce acceptable agreement with the observed x-ray diffraction pattern.

Finally, we examined the sensitivity of the *RDI* by shifting individual parameters separately from their optimal values of the full-filament overlap model (Fig. 11). It can be seen that the value of *RDI* was primarily affected by the azimuthal rotation angles (*φ_1_* and *φ_2_*) of crowns 1 and 2, the azimuthal rotation angle (*ε_p_*) of a myosin head (Fig. 11A) and the helical radius (*r_hp_*) of myosin crossbridges in the perturbed region (Fig. 11B). Thus, the structure of the perturbed region made a larger contribution to the diffracted intensities than that of the regular region since the perturbed region occupies ∼70% of the whole thick filament length (see Fig. 5A).

### Conclusions

Modeling of the myosin filaments of a typical higher vertebrate muscle has been undertaken using the x-ray diffraction pattern of resting frog sartorius muscles with nearly full thick-thin filament overlap from which partial lattice sampling effects had been removed. Inclusion of high-resolution myosin head structure with a pre-powerstroke shape and structural information concerning MyBP-C allowed trustworthy modeling of the x-ray diffraction data with sub-nanometer sensitivity. C-proteins had a slightly larger periodicity than myosin, consistent with EM analysis of cryo-sections, explaining how the projecting part of C-protein might interact with adjacent actin filaments in the A-band lattice of resting muscle. The interactions found between the heads of myosin crossbridges were basically similar to those of isolated myosin filaments observed by electron microscopic studies. The structure of the interacting heads along the myosin filament was different between in the regular region that lacks C-proteins and the perturbed region, covering somewhat more than the C-protein region, is affected by the presence of C-proteins. The “J-motif” configuration seen in invertebrate myosin was not an obvious feature of the interacting head motif in frog muscle. Although vertebrate striated muscle is primarily regulated by troponin-tropomyosin on the thin filaments, analysis of the resting structure of the thick myosin filaments revealed that the relaxed state might be also characterized by electrostatic interactions between the actin-binding region of one head and the converter domain of the other head of myosin crossbridges together with the possible interaction of C-protein and actin. Interacting head structures have now been observed in a variety of vertebrate and invertebrate striated muscles and presumably stabilize the relaxed state. Although the details of these interactions differ between muscle types, an interacting head-head structure appears to be a general motif for the resting myosin filaments whether the muscle is thick-filament regulated or thin-filament regulated, and it may be involved in cooperative interactions involved in regulation.
