# Correction: Head-Head Interactions of Resting Myosin Crossbridges in Intact Frog Skeletal Muscles, Revealed by Synchrotron X-Ray Fiber Diffraction

**DOI:** 10.1371/annotation/05f7579b-d2c6-43cd-b5b3-f883b4746474

**Published:** 2013-09-20

**Authors:** Kanji Oshima, Yasunobu Sugimoto, Thomas C. Irving, Katsuzo Wakabayashi

"C^o^-model" appears incorrectly throughout the manuscript. The correct text is "C^α^-model" 

